# Optimization of Plating Process on Inner Wall of Metal Pipe and Research on Coating Performance

**DOI:** 10.3390/ma16072800

**Published:** 2023-03-31

**Authors:** Chenming Zhang, Yongfeng Li, Xiaochang Xu, Mingming Zhang, Haoyuan Leng, Bin Sun

**Affiliations:** School of Mechanical and Electrical Engineering, Henan Institute of Science and Technology, Xinxiang 453003, China

**Keywords:** pipe inner wall, electro-brush plating, corrosion resistance, pipe, resistance capacity to deformation

## Abstract

An innovative brush plating process for preparing coatings on the inner wall of metal pipes is proposed, which aims to solve the limitations of current electroplating technology and improve the performance of the inner walls of metal pipes. While optimizing the process, the effect of working voltage on the microhardness, thickness, surface morphology, corrosion resistance, and elastoplasticity of the Ni coating on the inner wall of the tube was studied under the new process. The results indicate this technique can produce high-quality coatings on the inner wall of pipes in a simple and efficient manner. As the working voltage increases, the surface quality and comprehensive performance of the coating show an increasing trend followed by a decreasing trend. At 12 V, the coating exhibits the highest surface density and uniformity, the lowest surface roughness, the best corrosion resistance, and the maximum microhardness of 575.8 HV, with a corrosion current density of 1.040 × 10^−5^ A·cm^−2^, a corrosion rate of 0.122 mm·a^−1^, the maximum elastic recovery ratio h_e_/h_max_ of 0.36, and the best deformation resistance. This study demonstrated the effectiveness of this method in improving the durability and functionality of metal pipes and its potential for various industrial applications.

## 1. Introduction

As the global economic infrastructure develops rapidly, pipes, as essential tools for storing and transporting drinking water, oil and gas, and chemicals over long distances, have been extended annually [1]. However, corrosion and mechanical damage remain a significant threat during pipeline operation [2,3,4]. According to the European Gas Pipeline Incident Data Group (EGIG), the failure rate caused by corrosion damage in pipes has increased from 16.7% to 26.6% [5,6]. Moreover, global metal loss due to erosion–corrosion exceeds 100 million tons per year, accounting for 20% to 40% of the annual production, and corrosion can also cause severe pipeline accidents. On the other hand, in China, the annual loss caused by corrosion in water supply systems is approximately RMB 23.1 billion. If these issues are not effectively addressed in the long term, they will adversely affect the safety and reliability of pipeline systems, resulting in not only economic losses but also environmental impacts [7,8].

Numerous domestic and international scholars have conducted extensive research to solve the problem of pipe wall protection and repair [9,10]. Existing technologies for pipe wall protection and repair include spray coating, electrodeposition, hot-dip plating, and intercalated lining hose. Although the problem of pipe wall protection and repair has been improved to a certain extent, there are still certain limitations in practical applications. For example, the repair methods of intercalation and lining are prone to gaps between the repair material and the pipe, which may still be subjected to corrosion [11,12]. Commonly used spray protection technology also faces problems with uneven spraying and low bonding strength [13,14,15]. The quality of the coatings on the inner walls of long pipes is difficult to guarantee due to various factors, such as limitations in the length of the plating tank and the inability of the anode to reach the inside of the cathode pipe during electroplating. Some researchers have used plasma electroplating technology or special electrodeposition devices to prepare high-performance coatings on the inner wall of the pipe [9,16,17,18]. However, traditional electrochemical deposition still has certain limitations for depositing coatings on the inner walls of long pipes. In contrast, electro-brush plating has several advantages, including simple equipment without plating tanks, a flexible operation process, high current density, fast plating speed, stable plating solution, and good adhesion. Electro-brush plating has been widely used in the preparation of coatings due to these advantages [19,20,21,22,23,24,25,26,27]. In addition, due to the excellent corrosion resistance, oxidation resistance in room temperature air, and no reaction with concentrated nitric acid, nickel has been widely used in electro-brush plating protection, repair, and remanufacturing technologies [28,29,30,31,32,33]. Nickel-based metals are well suited to the requirements of this study for the protection and performance enhancement of the inner pipe wall.

In this study, an innovative process was proposed for preparing coatings on the inner walls of metal pipes, combining the technique of electro-brush plating. While optimizing the process, a Ni coating was prepared on the inner surface of the metal pipe by using a self-built in-pipe deposition platform, and the effect of the working voltage on the surface morphology and mechanical properties of the coatings was investigated. The results demonstrate the effectiveness of this method in enhancing the durability and functionality of metal pipes, and provide a new innovative processing method and technical foundation for the preparation of environmentally friendly, energy-efficient protective coatings on the inner walls of pipes [34,35,36].

## 2. Materials and Methods

### 2.1. Experimental Device and Process Flow

**Figure 1:**
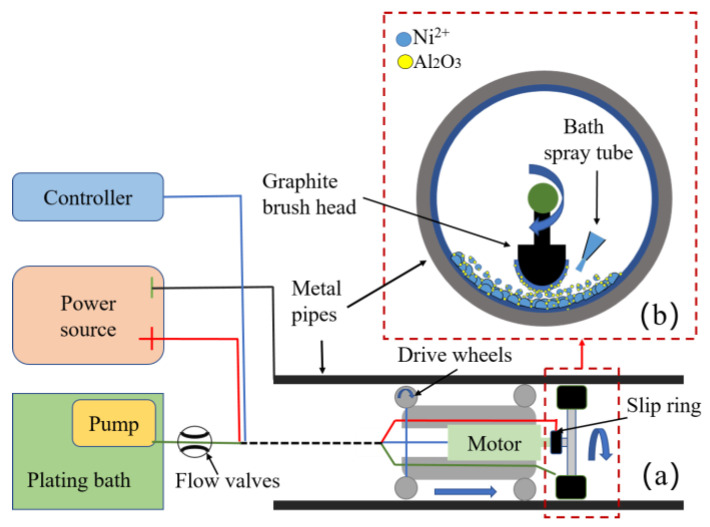
Pipe inner wall coating deposition system and coating growth diagram: (**a**) the pipe inner wall deposition system; (**b**) the processing area and coating growth mechanism.

A Ni coating was prepared on the inner wall of a pipe using a self-built experimental apparatus for in-pipe deposition. The apparatus comprises a control system, a pipe walking and driving device, an electrolyte supply system, a graphite brush head rotation device, and an electroplating power supply. The structure of the graphite brush head is designed as a semi-arc graphite block, and the brush head is wrapped with felt to facilitate filling with plating solution while adhering closely to the inner wall of the pipe, as depicted in Figure 1a. The brush head is connected to the anode of the power supply through a slip ring, and the front motor drives the brush head to rotate through a coupling. The apparatus is equipped with rubber driving wheels, a reduction driving motor at the wheel end, and a trailing cable at the tail end, enabling the experimental apparatus to travel inside the pipe and perform continuous electroplating under the control of the system. Figure 1a illustrates the schematic of the pipe inner wall deposition system, while Figure 1b depicts the processing area and coating growth mechanism.

Commercially available Q235 low-carbon steel pipe with an inner diameter of 80 mm and an outer diameter of 87 mm was used as the experimental substrate. Q235 low-carbon steel sheet with a size of 40 mm × 20 mm × 2 mm was used as the substrate for the optimization experiment of the pre-treatment process. The main process of the pre-treatment optimization test is as follows: grinding sample piece, pre-treatment, brush plating deposit, performance comparison. The process of pipe inner wall coating is as follows: lye degreasing, deionized water rinse, 5% HCl impurity removal activation, deionized water rinse, coating deposition, deionized water rinse, cut sample, deionized water rinse, drying, coating quality inspection.

### 2.2. Bath Composition and Process Conditions

In the pretreatment process optimization experiment, the configuration of the plating solution used for the 1st pre-treatment and 2rd pre-treatment is shown in Table 1.

The plating solution configuration shown in Table 2 was used in the trial production of the Ni coating on the inner wall of the pipeline and the experiment on the effect of voltage on the performance of the Ni coating. Prepare the electroplating solution at room temperature (25 °C), and stir for 15 min after preparation. After lye degreasing, the substrate was cleaned and activated with 5% HCl at room temperature (25 °C) until the surface of the substrate appeared grayish-white. Following activation, the inner walls of the pipe were electro-brush plated for 5 min to deposit the coating. The pipe was then cut to obtain samples, which were rinsed with deionized water and dried before conducting performance testing.

### 2.3. Experimental Design

The experiment was carried out at room temperature (25 °C), and the power supply was a MESTEK DP2050 DC power supply. The pre-treatment process optimization experiment was carried out on a flat electric brush plating platform, and the process parameters were as follows: working voltage 8 V, plating solution supply speed 16 mL/min, brush head moving speed 10 m/min; The process parameters remain unchanged, and the test plating experiments are carried out on the inner wall of the pipeline at 100 r/min, 180 r/min, and 260 r/min to verify the feasibility of the optimized process.

The preparation process parameters of the Ni deposition layer on the inner wall of the pipeline under different working voltages are as follows: plating solution supply speed 16 mL/min, brush head speed 180 r/min. Working voltages of 6 V (Label P-1), 8 V (Label P-2), 10 V (Label P-3), 12 V (Label P-4), 14 V (Label P-5), and 16 V (Label P-6) were used. The effect of working voltage on the surface morphology and properties of the coatings was investigated by varying the working voltage. Samples were taken from commercially available galvanized steel pipes of the same material and size (Label P) to participate in the tests and were compared. The enhancement effect of the electro-brush plating deposition process on the coatings on the inner walls of pipes was studied.

### 2.4. Test Instruments

(1) The surface morphology of the coating was analyzed using a Quanta 200 scanning electron microscope (Quanta 200, FEI Company, Hillsboro, OR, USA) at a magnification of 2000 times.
(2) The hardness of the deposited layer was characterized using a microhardness tester (VMH-002V, UHL, Asslar, Germany). A load of 25 gf was applied at 5 different positions for 15 s, and the average value of the obtained data were recorded.
(3) The cross-section of the coating was observed using a Leica DMi8 M metallographic microscope at a magnification of 500 times (Leica DMi8 M, Leica, Wetzlar, Germany).
(4) Electrochemical experiments were performed using an electrochemical workstation (CS2350, Wuhan Keist Instrument Co., Ltd., Wuhan, China) in a 3.5 wt% NaCl corrosion medium at room temperature. The three-electrode system had a saturated calomel electrode (SCE) as the reference electrode, a platinum electrode as the working electrode, and a potential range of 1500 mV to 1000 mV with a scan rate of 1 mV/s.
(5) The deformation resistance of the coating was analyzed using a G200 nanoindenter (KLA, Hayward, CA, USA). A load of 100 mN was applied to 5 different points on the surface of the coating, with a Poisson’s ratio of 0.25 set based on the material properties.

## 3. Results and Discussion

### 3.1. Process Optimization

#### 3.1.1. Optimization of Pretreatment Process and Bath Configuration

Traditional brush plating requires step-by-step electroplating and multiple pre-treatment processes, any of which are related to the final quality of the coating [37,38]. This cumbersome pre-treatment process is not suitable for the deposition of the inner wall of the pipes. The comparison of brush plating process steps with different pre-treatments is shown in Table 3.

**Figure 2:**
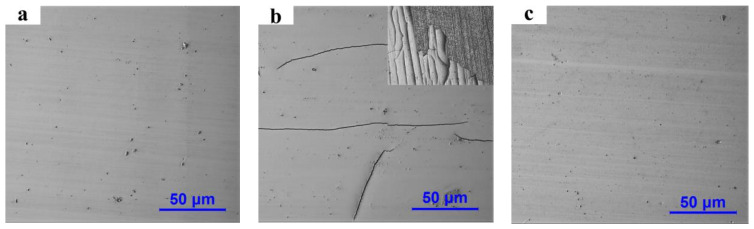
Surface morphology of coatings prepared by different pretreatment methods: (**a**) traditional brush plating pre-treatment; (**b**) pre-treatment 1; (**c**) pre-treatment 2.

Q235 steel plates with a size of 40 mm × 20 mm × 2 mm were selected as the substrate. Using identical process parameters and the bath configuration in Table 1, brush plating experiments were conducted using different pre-treatment techniques. The surface morphology of diverse samples, as portrayed in Figure 2. Figure 2a depicts the coating that has been deposited using the conventional brush plating pre-treatment method (first pre-treatment in Table 3). The coating’s surface exhibits a high degree of uniformity, with the exception of a few isolated protrusions and pores. The hardness of the coating is 420 HV. Figure 2b exhibits the coating that has been fabricated utilizing the technique expounded in 2rd pre-treatment of Table 3. This pre-treatment method adopts a lye degreasing and mixed acid activation procedure, to supplant the first seven steps of the conventional brush plating pre-treatment process, as indicated in Table 3. Figure 2b clearly shows the emergence of extensive cracks and peeling on the surface of the coating, resulting in unsatisfactory coating quality. These findings demonstrate that the quality of the electro-brush plating coating is significantly affected by the pre-treatment process, and the first seven pre-treatment steps cannot be blindly omitted in order to simplify the process. The first seven steps of pretreatment are not only for cleaning and activating the substrate, but more importantly, “Pre-nickel plating” as a transition layer can improve the adhesion and uniformity of the coating. This is the reason for the cracks in Figure 2b.

To solve this problem, enhancing the bonding strength of the coating is a prerequisite, such as replacing a more effective substrate activation method and a plating solution configuration with a better deposition effect. HCl can effectively remove the oxide film on the surface of the substrate, slightly etch the surface of the substrate to expose the metal crystal structure and better combine the metal ions with the surface of the substrate. Adding an appropriate amount of surfactant into the bath can reduce the surface tension of the bath and promote ion transfer, and it can provide better deposition conditions for coating deposition. Proven by experiment, pretreatment with 5% HCl can effectively activate the substrate. Figure 2c displays the coating prepared using the third pre-treatment and plating solution with surfactant, which has a bright and smooth surface without cracks and peeling, and a coating hardness of 445 HV, achieving a similar effect to traditional electro-brush plating processes.

#### 3.1.2. Trial Production of Pipe Inner Wall Coating after Process Optimization

**Figure 3:**
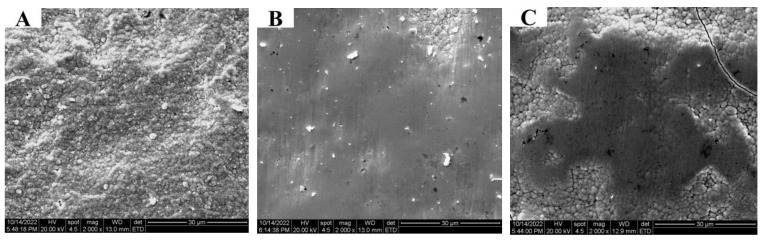
Surface topography of Ni coating on the inner wall of pipe trial-produced at different rotational speeds: (**A**) 100 r/min; (**B**) 180 r/min; (**C**) 260 r/min.

Using the plating solution configuration in Table 2, Ni coatings were trial produced on the inner wall of the pipe at different speeds based on the third pre-treatment process, as shown in Figure 3. In Figure 3A, the unit cell structure on the surface of the coating is severely coarsened, showing “cauliflower-like” grain clusters, and the hardness of the coating is 318 HV; in Figure 3B, the surface defects of the coating are reduced, uniform and smooth, the grain refinement effect is obvious, and the hardness of the coating is 367.5 HV; in Figure 3C, cracks appear on the surface of the coating, the size of the unit cell becomes larger, and the hardness of the coating is 354 HV. This is due to the fact that the current acting on the coating per unit area takes too long at low rotational speed, resulting in too fast a grain growth. However, high rotational speeds will lead to non-uniform current density during the deposition process and insufficient time for the plating solution to cover the entire surface uniformly, resulting in uneven deposition of the coating. At the speed of the brush head at 180 r/min, the deposition effect of the coating on the inner wall of the pipe is the best.

The above work has optimized the plating process, and the Ni coating has been trial-produced on the inner wall of the pipeline, the feasibility of the process has been verified. In the subsequent study, the effect of the working voltage on the surface morphology and properties of the coating on the inner wall of the pipeline was studied by using this process.

### 3.2. Surface Morphology of Pipe Inner Wall Coating under Different Voltage

**Figure 4:**
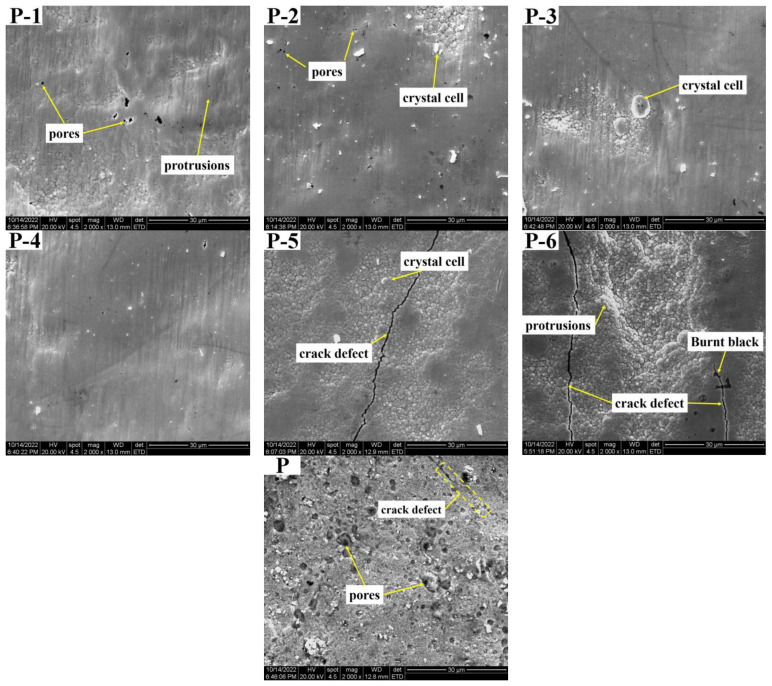
The corresponding surface topography of each sample: (**P-1**) 6V; (**P-2**) 8V; (**P-3**) 10V; (**P-4**) 12V; (**P-5**) 14V; (**P-6**) 16V; (**P**) galvanized pipe inner wall sample.

The surface morphology of the Ni coatings prepared on the inner wall of the pipe using the optimized process at different working voltages is shown in Figure 4, wherein the Label P is the inner wall coating morphology of the commercially available galvanized steel pipe as a comparison sample.

**Figure 5:**
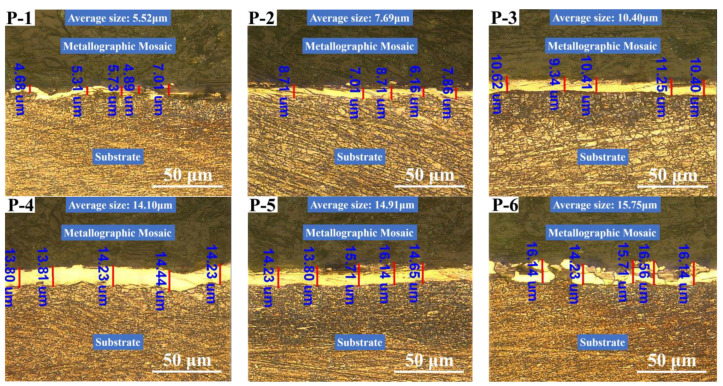
The corresponding section morphology and thickness of the coating prepared under different working voltages: (**P-1**) 6 V; (**P-2**) 8 V; (**P-3**) 10 V; (**P-4**) 12 V; (**P-5**) 14 V; (**P-6**) 16 V.

From the surface quality of the coatings, it can be observed that with the increase in the working voltage, the surface quality of the coating shows a trend of improvement first and then deterioration. When the working voltage is 12V, the coating surface shows most dense performance overall. With the gradual increase in the working voltage, the coating quality deteriorates. In Figure 4(P-5), the crystal grains begin to coarsen, and cracks gradually appear. As the working voltage reaches 16 V, the deterioration of the coating surface quality worsens, cracks become more severe, and even burning and blackening of the coating can be observed.

**Figure 6:**
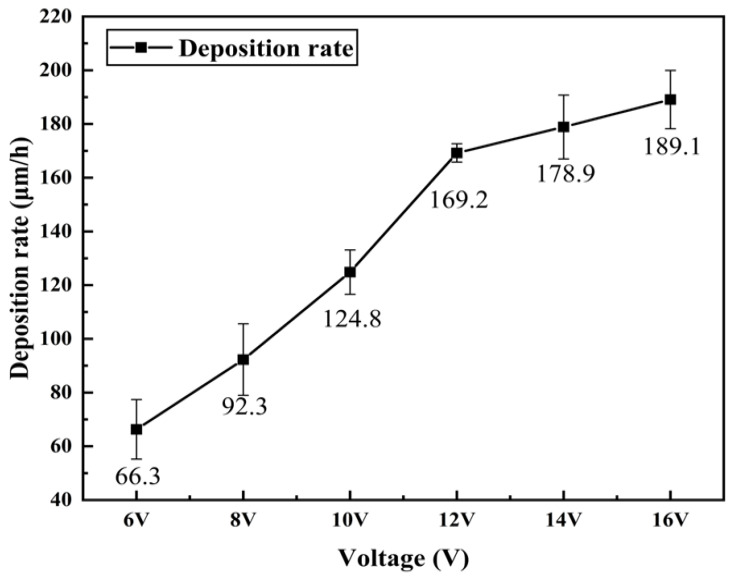
The deposition rate of the coating under different working voltage.

Because the surface topography of the nickel plating layer on the inner wall of the electro-brush plating pipe is greatly influenced by the working voltage, as demonstrated in Figure 4(P-1–P-6). The coating surface appears as typical “cauliflower-like” crystal cell grains with random distribution of defects such as protrusions and pores at a low working voltage. However, as the working voltage increases, the coating becomes more uniform and smooth, and the number of pores decreases. At a working voltage of 12 V, as shown in Figure 4(P-4), the coating surface is characterized by small protrusions and tiny pores in some locations with an overall smooth and dense appearance. This is due to the increase in current density resulting from an increase in working voltage, which leads to a more uniform distribution of metal ion charges on the surface [39]. As a result, the nucleation rate of crystal grains is greater than the growth rate, leading to a gradual decrease in grain size and, consequently, a dense, smooth, and less defective coating surface. During the electroplating process, hydrogen gas tends to attach to the coating surface, which can lead to the formation of pores. However, during the electro-brushing deposition process inside the pipe, the outer layer of the brush head is wrapped in elastic felt and maintains a constant relative motion with the cathode. This movement and friction during the electro-brush plating process effectively remove hydrogen gas from the coating surface and polish the cauliflower-like crystal cell grains that grow too quickly. As a result, the surface quality of the coating is greatly improved, becoming smooth and dense with fewer defects [38,40].

Compared with Figure 4(P-4), the crystal cell size in the coating in Figure 4(P-5,P-6) is significantly larger, the uniformity of the coating surface is reduced, and the porosity is increased. This can be attributed to the increase in current that occurs with high voltage during the electro-brushing process, which leads to the deposition of isolated globular crystals due to high overpotential and current density. Under these conditions, the crystal growth rate exceeds the nucleation rate, resulting in rapid crystal growth and roughness on the coating surface. Additionally, the rapid depletion of Ni ions and inadequate replenishment near the cathode can result in concentration polarization. The high working voltage also generates excess heat, accelerating the drying of the solution film on the coating surface and further exacerbating concentration polarization near the cathode. The excess voltage promotes hydrogen evolution, and many gas bubbles cannot be discharged in time, leading to the generation of internal porosity and increased internal stress in the coating. These factors collectively degrade coating quality and result in cracking, as well as more severe anode erosion, causing the coating to overheat and blacken, as observed in Figure 4(P-6).

Figure 4(P) reveals the presence of a large number of defects, such as pores, cracks, and pits, in the inner wall coatings of commercially available galvanized steel pipes. In contrast, the metal pipe inner wall coatings prepared using the electro-brushing technique at a working voltage of 12 V demonstrate superior surface quality, as demonstrated by Figure 4(P-4).

### 3.3. Cross-Sectional Morphology and Deposition Rate Analysis

The cross-sectional morphology and deposition rate of the Ni coatings prepared on the inner wall of the pipe using the optimized new process at different working voltages are shown in Figure 5 and Figure 6. In order to meet the simple and fast requirements in actual production and application as much as possible, the inner wall of the pipe was not polished before brush plating, so the bonding surface between the coating and the substrate is not straight, but fluctuates up and down following the defect trend of the substrate. The cross-sectional morphology of the coating appears after FeCl_3_ corrosion.

Figure 5 and Figure 6 demonstrate that the thickness and deposition rate of the coating increase proportionally to the gradual increase in the working voltage. At low working voltages, depicted in Figure 5(P-1,P-2), the current density passing through the unit area is insufficient to provide enough driving force for the coating deposition process, resulting in slow deposition and conspicuous defects at the interface with the substrate. As depicted in Figure 5(P-4), as the working voltage increases, the coating’s thickness and cross-sectional uniformity gradually improve, defects decrease, and adhesion to the substrate increases. However, when the working voltage surpasses a certain threshold, as shown in Figure 5(P-5,P-6), the coating quality deteriorates, the thickness becomes uneven, and a plethora of protrusions and depressions emerge on the coating surface. If the voltage surpasses a specific threshold, the coating becomes ablated and destroyed, leading to numerous cracks and even detachment. These results are consistent with those depicted in Figure 4(P-5,P-6).

This phenomenon can be explained as follows: As the working voltage increases, the unit area current increases, resulting in a higher deposition rate, which is directly proportional to the product of the current passing through the unit area and the duration of the electrolysis process. When the duration is fixed, a higher current passing through the unit area leads to a faster deposition rate. However, excessively high working voltage can cause concentration polarization and hydrogen evolution, which results in poor surface quality of the coating. The aggregation of a large number of crystal cells and grains also leads to a porous and loose coating, while some loosely bound grains may be removed by the motion of the brush head. This leads to the growth rate of deposition rate slows down and the deterioration of the cross-sectional quality of the coating after a working voltage of 12 V [41].

The results indicate that using an appropriate working voltage during the electro-brushing deposition process on the inner wall of the pipe can ensure that the coating exhibits good surface, cross-sectional morphology and is well-adhered to the substrate while still achieving a high deposition rate.

### 3.4. Hardness of Pipe Inner Wall Coating under Different Voltage

**Figure 7:**
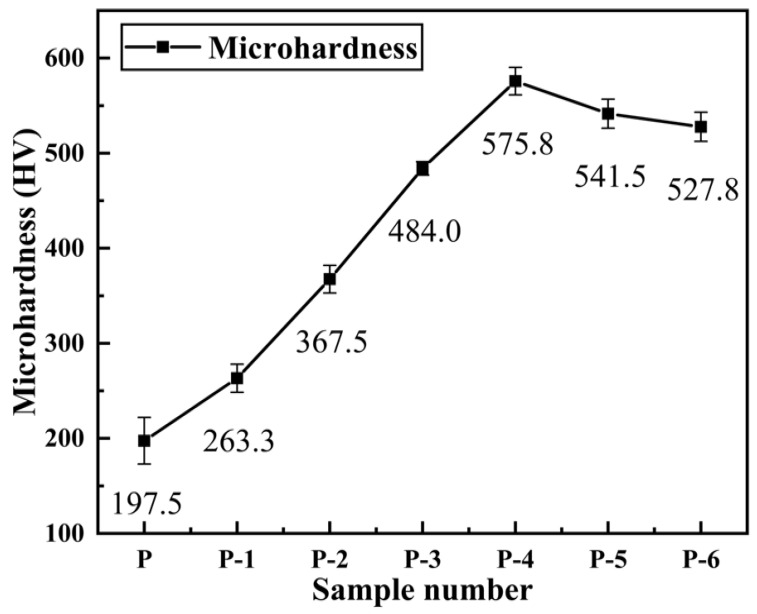
Microhardness corresponding to different samples: (P-1) 6 V; (P-2) 8 V; (P-3) 10 V; (P-4) 12 V; (P-5) 14 V; (P-6) 16 V; (P) galvanized pipe inner wall sample.

The hardness values corresponding to different samples are shown in Figure 7, wherein the label P is the hardness value of the inner wall coating of the commercially available galvanized steel pipe as a comparison sample.

It can be observed that the microhardness of the coating increases first and then decreases with the increase in working voltage. When the working voltage is 12 V (P-4), the hardness value reaches the maximum value of 575.8 HV. According to the surface morphology and cross-sectional morphology results shown in Figure 3 and Figure 4, the variation trend of coating hardness with operating voltage is related to the surface quality of the coating. As the working voltage is increased, the coating gradually becomes more uniform and dense, with a refined grain size and a smaller and more uniform structure, while defects such as air holes are reduced, resulting in an increase in hardness value. However, if the working voltage continues to increase beyond 12 V, an excessive voltage leads to the accumulation of a large number of crystal cells and grains, causing the growth rate of the grains to exceed the nucleation rate. Additionally, concentration polarization and hydrogen evolution reactions become more severe, leading to an increase in stress within the coating and the formation of defects such as pores and cracks, resulting in a decrease in hardness value. As the working voltage is increased to 14 V (P-5) or even 16 V (P-6), the surface of the coating displays defects such as cracks, air holes, and burning–blackening, causing a further reduction in hardness value.

According to the Hall–Petch formula [42]:

*σ*_*S*_ = *σ*_0_ + *Kd*^−1/2^ (1)

where *σ*_*S*_ represents the yield strength, and *σ*_0_ and *K* are constants, while *d* is the average grain diameter in a polycrystalline material. It is evident that the surface of the coating becomes denser with a smaller grain size, leading to an increase in the microhardness of the coating. Consequently, the hardness value of the coating is closely related to its surface quality. A coating with a uniform and dense surface, obtained at an optimal working voltage, exhibits a higher hardness value.

The results show that the Ni coating on the inner wall of the pipe prepared by brush plating at a suitable working voltage has high microhardness. Additionally, its hardness is obviously better than the inner wall coating of commercially available galvanized steel pipes.

### 3.5. Corrosion Resistance of Pipe Inner Wall Coating under Different Voltage

**Figure 8:**
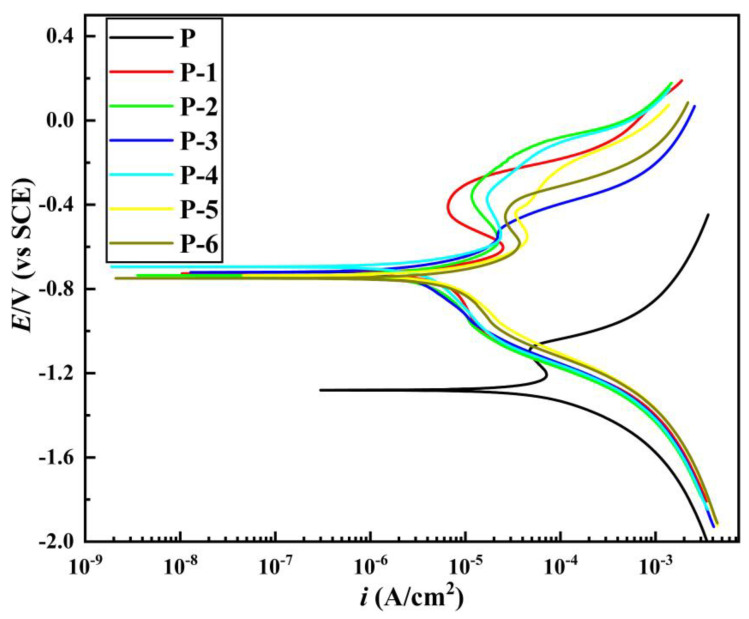
The potentiodynamic polarization curves of different samples in 3.5 wt% NaCl solution.

The corrosion resistance of the coatings prepared under different voltages was evaluated. Figure 8 displays the corrosion polarization curves obtained by dynamic potential scanning of different samples in a 3.5% NaCl solution. The fitting results of the polarization curves are shown in Table 4.

From the results presented in Figure 8 and Table 4, as the working voltage increases, the corrosion potential initially increases and then decreases, while the corrosion current density decreases and then increases. The gradual increase in the corrosion resistance of the coating is reflected by the positive shift of the corrosion potential and the negative shift of the corrosion current. According to Table 4, the maximum corrosion potential and minimum corrosion current density are observed at a working voltage of 12 V (Label P-4), corresponding to −0.6945 V and 1.040 × 10^−5^ A·cm^−2^, respectively. The corresponding corrosion rate is 0.1220 mm·a^−1^, indicating the minimum corrosion tendency of the coating. A further increase in the working voltage leads to a decrease in the corrosion potential and an increase in the corrosion current, which ultimately reduces the corrosion resistance of the coating.

This is because the corrosion resistance of the coating is closely related to the surface quality of the coating. At lower working voltages, the coating surface displays noticeable protrusions and pores (as illustrated in Figure 4(P-1,P-2)), which create pathways for the diffusion of corrosive solutions, resulting in decreased corrosion performance. As the working voltage is gradually increased, the coating surface becomes increasingly uniform and dense, reaching its optimal structure at 12 V, characterized by a uniform and fine structure, a prominent grain refinement effect, minimal roughness, and the lowest number of defects. At this point, the corrosion resistance of the coating is the best. This is because an increase in working voltage leads to a corresponding increase in unit area current, which promotes a more uniform distribution of metal ion charge on the surface, resulting in a higher nucleation rate of grains compared to their growth rate and, consequently, leading to a decrease in grain size. Another reason is that the relative motion between the brush and the cathode can help to discharge the hydrogen gas on the surface of the coating, and it can also polish and refine the coarse and loose grains of the coating surface. After the working voltage exceeds 12 V, the excessive voltage causes a large accumulation of grains and crystals, and the surface of the coating becomes rough. At the same time, the hydrogen evolution reaction intensifies, the internal stress of the coating increases, the porosity of the coating surface increases significantly, and cracks appear (Figure 4(P-5,P-6)). The corrosive medium enters the active area inside the coating through these cracks, corroding the coating and reducing its corrosion resistance [41,43,44].

As the working voltage increases, the corrosion potential shows a trend of increasing first and then decreasing. An elevation in the corrosion potential of a coating typically indicates an increase in its corrosion resistance. When the corrosion potential of the coating moves in a positive direction, it signifies a decrease in its electrochemical activity and an enhancement in its corrosion resistance. This is because an elevated corrosion potential can reduce the dissolution rate and suppress the corrosion reaction of the metal. However, the positive shift in the corrosion potential of the coating is the result of multiple factors. The main reason is that, with the gradual increase in the working voltage of brush plating, the deposition rate also increases correspondingly. As a result, the thickness of the coating increases, and the corrosion resistance gradually improves. The current density in the brush plating process also increases with the increase in the working voltage, which can make the grains of the coating more dense, reduce the entry of oxygen, water, and other corrosive substances, thereby improving the corrosion resistance of the substrate. Additionally, increasing the working voltage of brush plating can improve the density and uniformity of the coating, further enhancing corrosion resistance. However, when the voltage exceeds 12 V, the corrosion potential begins to move in the negative direction, which is caused by the deterioration of the coating quality resulting from the voltage exceeding the threshold. In summary, the positive shift in the corrosion potential of the deposited layer is a comprehensive result of the increase in deposition rate, grain density, and coating uniformity.

The results indicate that the inner coating of commercially available galvanized steel pipes exhibits a high corrosion rate. In contrast, the nickel coating deposited on the inner surface of the pipe at a working voltage of 12 V demonstrates superior corrosion resistance, exhibiting significantly better performance compared to the inner coating of commercially available galvanized steel pipes.

### 3.6. Elastic-Plastic Deformation Capacity of Pipe Inner Wall Coating under Different Voltage

**Figure 9:**
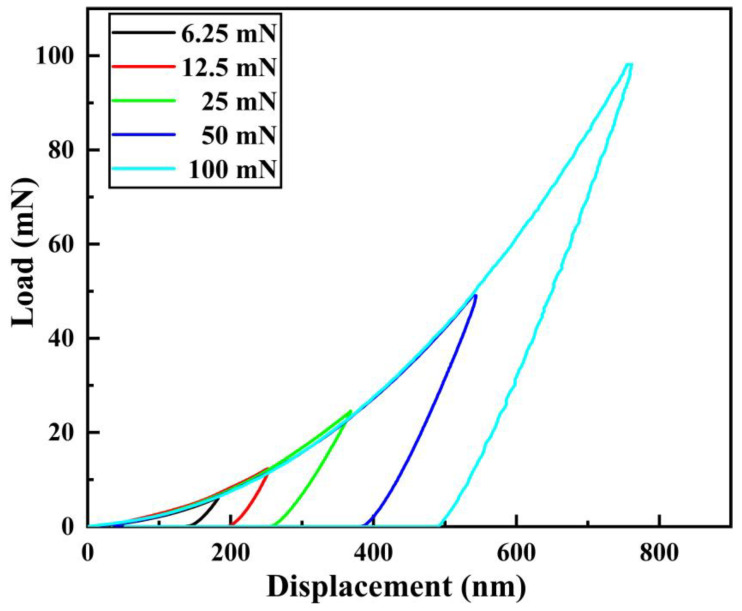
Displacement–load curves of sample P-4 under different loads.

Under different loads (6.25 mN, 12.5 mN, 25 mN, 50 mN, 100 mN), indentation tests were carried out on the Ni coating of the P-4 sample. Among them, the measured displacement–load curve of sample P-4 is shown in Figure 9. It can be seen from Figure 9 that the loading curves of Ni coatings under different loads have a good coincidence degree, and the unloading curves are at regular intervals, indicating that the experiment has good repeatability, and the data are reliable.

**Figure 10:**
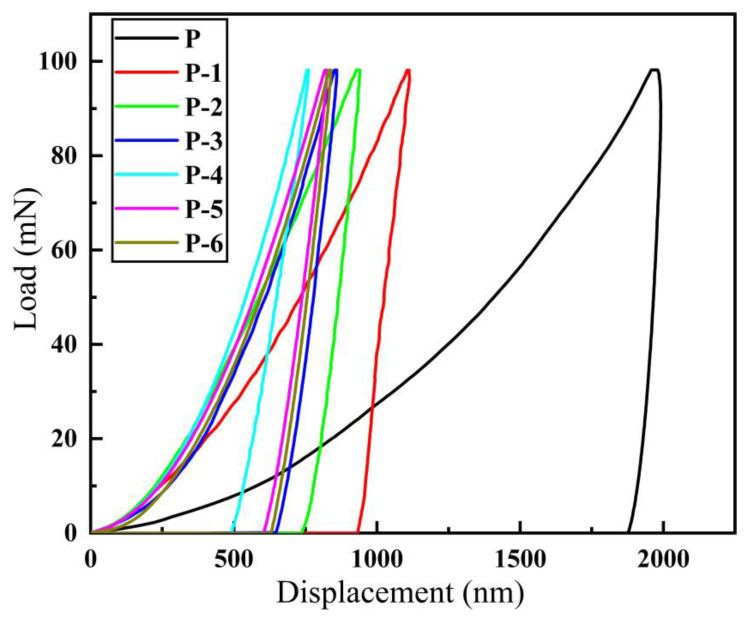
Displacement–load curve of different samples: (P-1) 6 V; (P-2) 8 V; (P-3) 10 V; (P-4) 12 V; (P-5) 14 V; (P-6) 16 V; (P) galvanized pipe inner wall sample.

**Figure 11:**
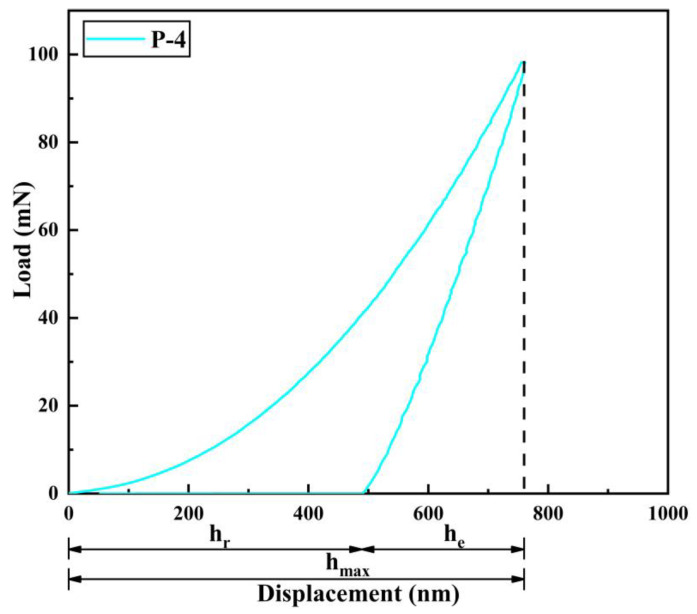
Schematic diagram of the relationship between h_e_, h_r_ and h_max_.

**Figure 12:**
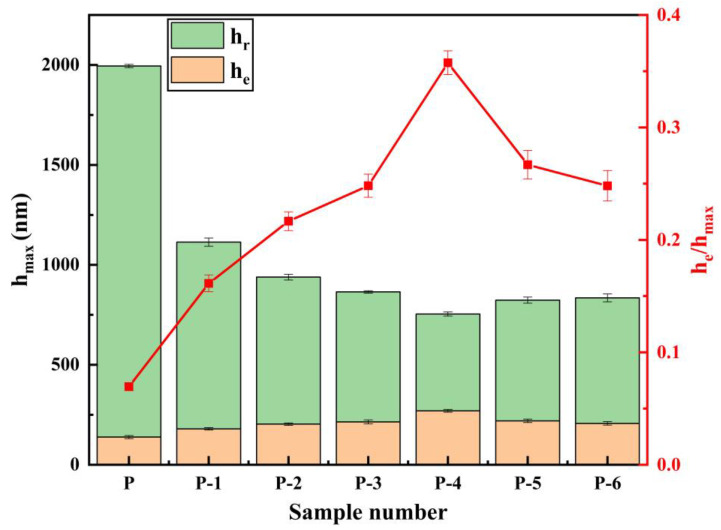
The trend of maximum displacement and elastic recovery rate (h_e_/h_max_) corresponding to different specimens.

Under the same load (100 mN), indentation tests were carried out on five different parts of each sample, and reliable experimental data were selected. Additionally, the displacement–load curves obtained from the tests are presented in Figure 10. Figure 11 illustrates the relationship between maximum indentation depth (h_max_), residual depth (h_r_), and elastic recovery depth (h_e_) in the indentation experiments. Each sample corresponds to the elastic recovery depth (h_e_), residual depth (h_r_), and maximum indentation depth (h_max_), as well as the elastic recovery rate curve, as shown in Figure 12.

The displacement–load curves for all samples are depicted in Figure 10. The absence of any convex or jitter phenomena in the curve during the experimental loading and unloading process indicates that the experiment was not subject to abnormal interference and that the results are reliable. As the working voltage increases, the indentation curve initially shifts to the left and subsequently to the right, while the maximum indentation depth (h_max_) declines and then rises. Meanwhile, the elastic recovery rate increases initially, and then decreases. The coating’s toughness increases as the elastic recovery rate grows, and its plasticity is directly proportional to the ratio of elastic recovery depth (h_e_) to maximum indentation depth (h_max_). At a working voltage of 12 V (Label P-4), the curve reaches the far left, with h_max_ reaching a minimum of 760 nm, h_e_ reaching a maximum of 271 nm, and the elastic recovery ratio h_e_/h_max_ reaching a maximum of 0.36. At this point, the coating exhibits its best elastic-plastic performance.

The reason for this is that, as the operating voltage gradually increases, the current density per unit area increases, causing the distribution of metal ion charges to become more uniform and the grain size to decrease. The movement of the brush head relative to the cathode facilitates the removal of air bubbles from the coating surface, and at the same time, it can polish the raised unit cells to refine the grains. This dynamic process results in a more compact and uniform surface, and an enhancement in its resistance to deformation.

**Figure 13:**
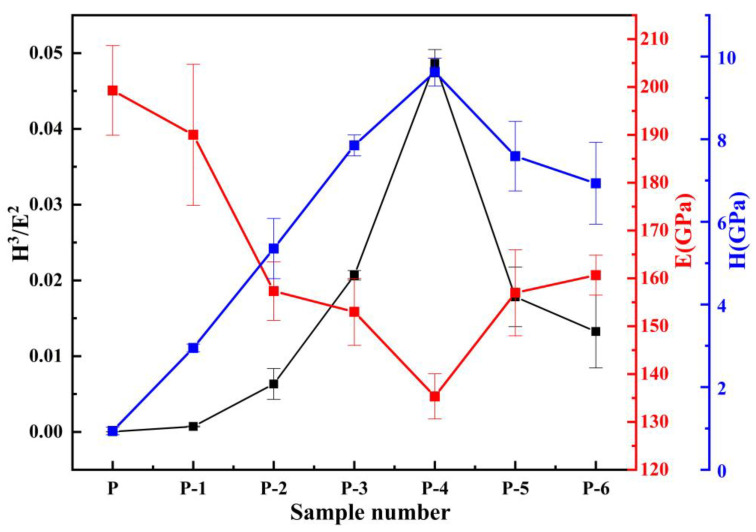
Trends in microhardness (H), Young’s modulus (E), and ratio (H^3^/E^2^) of different specimens.

The trends in microhardness (H), Young’s modulus (E), and the ratio (H^3^/E^2^) for each sample are illustrated in Figure 13. The experimental findings reveal that during the indentation test, the microhardness initially increases and then decreases as the operating voltage increases. The maximum value of microhardness is obtained at a working voltage of 12 V, which is consistent with the Vickers hardness test results. Young’s modulus (E) is a material property that characterizes the physical quantity and is solely related to the material’s properties. The ratio of microhardness (H) to Young’s modulus (E) (H^3^/E^2^) can also be utilized to evaluate the coating’s fracture toughness. The greater the ratio, the stronger the resistance to deformation. It can be observed from Figure 12 that the ratio of H^3^/E^2^ initially increases and then decreases as the working voltage increases, which is consistent with the elastic recovery rate trend. The ratio of H^3^/E^2^ reaches its highest value of 0.049 at a working voltage of 12 V, indicating the best resistance to plastic deformation [45].

As evidenced by Figure 10 and Figure 12, the indentation curve of the inner wall coating (Label P) of the commercially available galvanized steel pipe is on the far right, and the deformation resistance and elastic recovery rate are the worst. Consequently, the coating exhibits the poorest elastic-plastic performance. Based on the findings, the pipe inner wall nickel coating fabricated under a 12 V working voltage demonstrates favorable elastic-plasticity and resistance to deformation. Furthermore, the inner wall coating produced by this method exhibits significantly better elastic-plasticity compared to the commercially available galvanized steel pipe inner wall coating.

## 4. Conclusions

A new process for electro-brush plating deposition on the inner wall of metal pipes is proposed. Using the optimized new process, a Ni coating with good performance was successfully prepared on the inner wall of the metal pipe, and the influence of working voltage on the surface morphology and mechanical properties of the coating was studied. It proves the effectiveness of this method in improving the durability and functionality of metal pipes, and provides a new innovative processing method and technical basis for the preparation of environmentally friendly, high-efficiency, and energy-saving protective coatings on the inner wall of pipes.

(1) Through the optimization of the pre-treatment process and plating solution formula, nine steps in the electro-brush plating process have been simplified to five steps. Moreover, Ni coatings with dense and uniform surface and good performance can be prepared conveniently and efficiently on the inner wall of metal pipes.
(2) As the working voltage gradually increases, the surface and cross-sectional morphology of the pipe inner wall coating exhibit a trend of improvement followed by deterioration. The deposition rate shows a trend of acceleration followed by deceleration. By using an appropriate working voltage (12 V), a coating with superior surface and cross-sectional morphology, as well as good adhesion to the substrate, can be obtained while maintaining a high deposition rate (169.2 μm/h).
(3) The microhardness, corrosion resistance, and elastic-plasticity of the Ni coating prepared on the inner wall of the pipe show a trend of initially increasing and then decreasing as the working voltage gradually increases. The coating exhibits the best surface quality, the fewest defects, and the most significant grain refinement effect when the working voltage is 12V. At this voltage, the coating has a hardness of 575.8 HV, a corrosion current density of 1.040 × 10^−5^ A·cm^−2^, a corrosion rate of 0.122 mm·a^−1^, and an elastic recovery ratio (h_e_/h_max_) of 0.36. The results indicate that the coating prepared at 12 V has the best microhardness, corrosion resistance, and deformation resistance.
(4) Ni coating on the inner wall of a metal pipe via an optimized electro-brush plating deposition process. Compared with the coating on the inner wall of commercially available galvanized steel pipes, the resultant Ni coating exhibited significant enhancements in surface morphology, coating hardness, corrosion resistance, and elastoplasticity. Moreover, the proposed method features several advantages including simple and convenient equipment, uniform and dense surface morphology, high deposition rate, and simplified plating solution and process.

## Figures and Tables

**Table 1:** Optimize the configuration of the plating solution in the pretreatment process.

Bath Compositions	Concentration	Depositing Parameters	Values
NiSO_4_·_6_H_2_O	250 (g·L^−1^)	Temperature	25 (°C)
C_2_H_7_NO_2_	45 (g·L^−1^)	pH	6.5–7.5
(NH_4_)_3_C_6_H_5_O_7_	35 (g·L^−1^)	Brush head speed	10 (m/min)
C_2_H_8_N_2_O_4_·H_2_O	1 (g·L^−1^)	Time	5 (min)
NH_3_·H_2_O	100 (mL/L)	working voltage	8 V
		Bath supply speed	16 (mL/min)

**Table 2:** The composition of the brush plating solution for the inner wall of the pipe.

Bath Compositions	Concentration	Depositing Parameters	Values
NiSO_4_·_6_H_2_O	250 (g·L^−1^)	Temperature	25 (°C)
C_2_H_7_NO_2_	45 (g·L^−1^)	pH	6.5–7.5
(NH_4_)_3_C_6_H_5_O_7_	35 (g·L^−1^)	Brush head speed	100,180,260 (r/min)
C_2_H_8_N_2_O_4_·H_2_O	1 (g·L^−1^)	Time	5 (min)
NH_3_·H_2_O	100(mL/L)	working voltage	6 V, 8 V, 10 V, 12 V, 14 V, 16 V
Surfactant	0.1 (g·L^−1^)	Bath supply speed	16 (mL/min)

**Table 3:** Comparison of brush plating process steps with different pre-treatments.

	First Pre-Treatment	Second Pre-Treatment	Third Pre-Treatment
Step	Operation	Operation	Operation
1	Electroclean	Lye degreasing	Lye degreasing
2	Rinse	Rinse	Rinse
3	Activate 1	Mixed acid activation (15%HNO_3_ + 15% H_3_PO_4_)	Impurity removal and activate (5%HCl)
4	Rinse	Rinse	Rinse
5	Activate 2	Pure Ni plating	Pure Ni plating
6	Rinse		
7	Pre-nickel plating		
8	Rinse		
9	Pure Ni plating		

**Table 4:** Important polarization curve parameters.

Number	Corrosion Potential/V	Corrosion Current Density/A·cm^−2^	Corrosion Rate/mm·a^−1^
P	–1.281	7.651 × 10^−5^	0.898
P-1	–0.727	3.615 × 10^−5^	0.424
P-2	–0.735	1.749 × 10^−5^	0.205
P-3	–0.721	1.262 × 10^−5^	0.148
P-4	–0.695	1.040 × 10^−5^	0.122
P-5	–0.735	2.589 × 10^−5^	0.304
P-6	−0.749	3.107 × 10^−5^	0.364

## Data Availability

Not applicable.
